# Procedure and Aortic Remodeling Effects of Entry Closure with Stentgraft for Type B Aortic Dissection: Comparison between the Patients with Narrow True Lumen and Those with Aneurysmal Dilated False Lumen

**DOI:** 10.3400/avd.oa.22-00089

**Published:** 2022-09-25

**Authors:** Atsushi Aoki, Kazuto Maruta, Tomoaki Masuda, Tadashi Omoto

**Affiliations:** 1 Division of Cardiovascular Surgery, Department of Surgery, Showa University, Tokyo, Japan

**Keywords:** type B aortic dissection, patent false lumen, stentgraft, narrow true lumen, dissecting aortic aneurysm

## Abstract

**Objectives**: Appropriateness of device selection, procedure protocol and aortic remodeling effects of entry closure (TEVAR) with stent-graft (SG) for patent false lumen type B aortic dissection (TBAD) were compared between the patients with narrow true lumen (narrow group) and those with aneurysmal dilated false lumen (aneurysmal group).

**Methods**: Twenty-six patients with narrow true lumen (narrow group) and 20 patients with aneurysmal false lumen (aneurysmal group) were included in this study. In narrow group, straight SG was implanted from Zone 3 regardless the distance between the left subclavian artery and entry. In aneurysmal group, straight or taped SG was implanted with proximal landing zone length 20 mm or more. Thoracic aortic anatomy was evaluated by CT and aortic remodeling was defined as true lumen diameter ≥50% of the aortic diameter and occlusion of false lumen. Aorta related death, retrograde type A aortic dissection (RTAD), stentgraft induced new entry (SINE) and aortic maximum diameter enlargement 5 mm or more (aortic expansion) were included in the aortic event.

**Results**: There was no procedure related complication in narrow group and 1 patient died due to aortic rupture in aneurysmal group, Type Ia endoleak by enhanced CT 7 days after TEVAR was detected in one patient in each group. Achievement of aortic remodeling was significantly better in narrow group. Aortic event occurred in only one patient in narrow group, in whom aortic expansion was observed. In aneurysmal group, aortic event occurred 12 patients (60%) and 2 RTAD, 5 SINE, and 8 aorta expansion were observed. Aortic event free rate was significantly better in narrow group.

**Conclusion**: TEVAR procedure for the TBAD patients with narrow true lumen seemed to be appropriate, however, different TEVAR procedure or additional procedures would be required for those with aneurysmal dilated false lumen to obtain favorable outcomes. (This is secondary publication from Jpn J Vasc Surg 2021; 30: 347–357.)

## Introduction

The principle of stent-graft (SG) therapy for aortic disease is a healthy-to-healthy procedure, in which SG should be placed between normal aorta proximal and distal to aortic disease, and this heathy-to-healthy procedure is essential to achieve a favorable outcome. Entry closure using SG (TEVAR) for Type B aortic dissection (TBAD) with a patent false lumen is contradictory to this principle of SG therapy, because dissection remains in the distal landing zone at least at the time of SG implantation. However, when aortic remodeling, such as false lumen disappearance, is achieved by TEVAR procedure, the aortic anatomy in the proximal and distal landing zones normalizes, thereby conforming to the principle of SG treatment and favorable outcome might be achieved with TEVAR.

Since 2012, we have been performing TEVAR for the patients with TBAD without complications and for the patients with residual dissection after the surgery for acute Type A aortic dissection, if minimum true lumen (TL) diameter was 50% or less of the aortic diameter in the thoracic aorta at the onset of TBAD or during the follow-up (narrow TL groups). Also, we performed TEVAR for the patients referred to our hospital because maximum aortic diameter became 50 mm or more due to false lumen dilation (aneurysmal group). In this study, we attempted to validate the appropriateness of TEVAR procedure for both groups, and to compare the aortic remodeling effects of TEVAR between the two groups.

## Subjects and Methods

At our hospital, SG was implanted in the thoracic aorta in 182 patients from October 2012 to May 2021, of these, 93 received SG implantation for TBAD or residual dissection of the descending aorta after the surgery for Type A aortic dissection. Of these 93 patients, 29 with an ulcer-like projection (ULP), 11 with true aneurysm, 3 with a rupture, and 4 with an additional SG implantation following TEVAR for TBAD were excluded from the study. Consequently, 46 patients, including 26 with a minimum TL width of 50% or less of the aortic diameter in the thoracic aorta (narrow TL group) and 20 with dissecting aortic aneurysm with a maximum aortic diameter of 50 mm or more (aneurysmal group), were included in this study.

The preoperative anatomy of the descending aorta was evaluated by the aortic diameters and TL diameter (the long and short axes) at the levels of the proximal entry end, the pulmonary artery bifurcation, 12th thoracic vertebra, and minimum TL short axis.

Moreover, in 11 patients of the aneurysmal group whose computed tomography (CT) data within 5 years of onset were available, the anatomy of the descending aorta was evaluated at an average of 19 months after the onset and before TEVAR (112 months after the onset). The average interval between two CT procedures was 56 months. The proximal neck length was measured as the distance from the left common carotid artery to the proximal entry in Zone 2 TEVAR and the distance from the left subclavian artery to the proximal entry in Zone 3 TEVAR. The proximal neck diameter was measured as the aortic diameter just distal to the left common carotid artery in Zone 2 TEVAR and the aortic diameter just distal to the left subclavian artery in Zone 3 TEVAR. The short and long diameter of TL was measured at the entry site and TL diameter at the entry site was defined as the average of short and long diameter.

TEVAR was performed under general anesthesia in an operating room using a mobile digital subtraction angiographic system (ARCADIS Avantec, Siemens, Erlangen, Germany) or in a hybrid operating room equipped with Artis Zeego (Siemens, Erlangen, Germany).

Regarding the size of the SG, approximately 120% oversizing was selected for both groups when the proximal landing zone was artificial graft, and 105%–115% and 110%–120% oversizing was selected for the narrow TL and aneurysmal groups, respectively, when the proximal landing zone was native aorta. According to the selection of straight or tapered SGs, the straight type was generally selected for the narrow TL group even when the ratio of the SG diameter to the TL diameter at the end of SG (distal oversizing) was more than 200%. However, for the aneurysmal group, the tapered type was also selected depending on the average TL diameter in the distal landing zone in order to maintain distal oversizing below 200%. The device type was selected for both the groups with the aim of minimizing the possibility of anterograde flow into false lumen (Type Ia endoleak) based on the anatomy of the proximal landing zone, and the device with a bared SG on the proximal side was accepted. SG was implanted from Zone 3 in the narrow TL group, regardless of the distance from the left subclavian artery to the entry. In contrast, for the aneurysmal group, ≥20 mm distance was secured between the entry and the SG proximal edge, and the SG was implanted from either Zone 1 or 2, as applicable. For both groups, proximal balloon attachment of SG was performed when SG was implanted into the artificial graft. The endpoint of TEVAR during procedure differed between two groups. In aneurysmal group, the endpoint was defined as the disappearance of Type Ia endoleak throughout the study period and proximal balloon attachment or additional SG implantation was applied when a Type Ia endoleak was observed. Until 2015, the endpoint and procedure for Type Ia endoleak was the same in narrow TL group. Since 2016, the endpoint of TEVAR has been redefined as the dilation of the TL only in the narrow TL group and no additional procedure was done when true lumen addition was confirmed after SG deployment, even if Type Ia endoleak was detected.

CT follow up program was as follows: a contrast-enhanced CT after 7 days and 6 months postoperatively. Thereafter, either contrast-enhanced or plain CT was performed once a year. Postoperative contrast-enhanced CT was evaluated in early and delayed enhancement patterns. When contrast effect from the SG proximal side to the false lumen did not continue with contrast enhanced distal false lumen in delayed enhancement patterns, it was considered as a definitive Type Ia endoleak (definitive Ia). In contrast, when contrast effect from SG proximal side to the false lumen continued with the contrast enhanced distal false lumen in delayed enhancement patterns, it was considered a possible Type Ia endoleak (possible Ia).

The anatomy of proximal landing zone was evaluated by the proximal diameter ration (the ratio of TL diameter of the proximal edge of the entry to the proximal neck diameter) preoperatively and 1 week after TEVAR. The anatomy of distal landing zone was evaluated by distal oversizing (the ration of SG diameter to TL diameter at the end of SG) and the presence or disappearance of a false lumen. The anatomy of false lumen was evaluated by presence or absence of contrast enhancement, presence of disappearance of false lumen and true lumen ratio (the ratio of true lumen short axis diameter to the aortic diameter) at the proximal neck area, bifurcation of the pulmonary artery bifurcation, distal end of SG, and 12th thoracic vertebra. Aortic remodeling was defined as the TL ratio in the thoracic aorta was ≥50% and the contrast effect in the false lumen in the thoracic aorta or the false lumen had disappeared. Aortic event was defined as aorta-related deaths, retrograde Type A aortic dissections (RTAD), SG-induced new entry (SINE), and dilation of the maximum aortic diameter of 5 mm or more during follow-up.

For the statistical analysis, JMP Pro16 software (SAS Inc., Cary, NC, USA) was used. Chi-square or Fisher’s exact test were applied for category variables, and Wilcoxon test was applied for continuous variables. Hazard ratios of <0.05 were considered significant. The disappearance rate of the false lumen at distal landing zone, achievement rate of aortic remodeling, and aortic events free rate were analyzed using log-rank test based on the Kaplan–Meier method. This study was conducted in accordance with the Declaration of Helsinki after receiving approval from the Research Ethics Committee of Showa University (acceptance No.: 3125).

## Results

Regarding the patients’ background, no significant differences were observed in age, gender, and body physique between 2 groups. Moreover, there were no significant differences in the number of patients with surgical history for Type A aortic dissection (narrow TL group: arch replacement in six patients; aneurysmal group: ascending aortal replacement in one patient, arch replacement in six patients, and descending aortal replacement in one patient). The duration from the onset of TBAD to TEVAR was significantly shorter in the narrow TL group, with all patients undergoing TEVAR within 1 year of the onset. In contrast, 30% patients in the aneurysmal group underwent TEVAR after 5–10 years of onset and 40% underwent TEVAR after ≥10 years of onset. No significant differences were observed in the entry position between the two groups. The aortic diameter and the TL long axis diameter were significantly larger in the aneurysmal group at all levels. In contrast, no significant differences were observed in the TL short axis between the two groups (**Table 1**).

**Table 1:** Table 1 Patient back grounds and preoperative thoracic aortic anatomy

	Narrow TL	Aneurysmal	p value
	n=26	n=20	
Age (years)	59.6±10.0	64.4±10.0	0.1151
Female gender	8 (30.8%)	5 (25.0%)	0.6656
Height (cm)	165±10	164±9	0.5713
Weight (kg)	67.0±12.6	69.1±18.6	0.8680
Body surface area (m^2^)	1.73±0.20	1.74±0.26	0.8767
BMI	24.5±3.6	25.5±4.9	0.5062
History of grafting for thoracic aorta	6 (23.1%)	8 (40.0%)	0.2171
Period from onset to entry closure (months)	2.3±2.8	106.5±69.3	<0.0001
<1 year	26 (100%)	2 (10.0%)	
1–5 years	0	4 (20.0%)	
5–10 years	0	6 (30.0%)	
>10 years	0	8 (40.0%)	<0.0001
Primary entry position			
Major curvature	17 (65.4%)	10 (50.0%)	
Minor curvature	5 (19.2%)	5 (25.0%)	
The descending aorta	4 (15.4%)	5 (25.0%)	0.5999
Diameter of the descending aorta (mm)			
Proximal edge of entry	33.4±4.3	46.1±11.0	<0.0001
Pulmonary artery bifurcated level	35.2±7.2	49.7±8.5	<0.0001
12^th^ thoracic vertebrae	29.4±3.4	37.1±8.4	0.0006
Minimum true lumen width level	31.9±4.8	47.4±13.3	<0.0001
Minor axis of the true lumen (mm)			
Proximal edge of entry	14.4±4.4	16.0±4.9	0.5186
Pulmonary artery bifurcated level	11.7±4.4	12.7±3.3	0.3590
12^th^ thoracic vertebrae	10.3±2.7	11.0±2.7	0.4015
Minimum true lumen width level	6.6±2.6	8.2±2.2	0.0548
Major axis of the true lumen (mm)			
Proximal edge of entry	27.6±3.9	34.5±6.1	0.0003
Pulmonary artery bifurcated level	24.6±4.1	31.4±6.2	0.0008
12^th^ thoracic vertebrae	21.8±3.3	26.3±3.2	<0.0001
Minimum true lumen width level	22.2±5.2	28.6±4.5	0.0002

Patient backgrounds did not differ between two groups except the period from onset to entry closure was significantly longer in the aneurysmal group. Diameter of the descending aorta and major axis of the true lumen were significantly larger in aneurysmal group, however, minor axis of the true lumen did not differ between two groups in all levels.

In 11 patients in the aneurysmal group, changes in the diameter of the descending aorta were evaluated based on CT data within 5 years of onset and those before TEVAR surgery, which was performed at an average of 56 months after the previous CT session. The aortic diameter and the TL long axis increased significantly, except for those at the entry position. However, no significant differences were observed in the TL minor axis at any level (**Table 2**).

**Table 2:** Table 2 Change of the thoracic aortic anatomy in 11 patients of Aneurysmal group

	Within 5 years	Pre-TEVAR	p value
Diameter of the descending aorta (mm)			
Proximal edge of entry	43.2±5.4	44.5±11.9	0.3157
Pulmonary artery bifurcated level	40.1±5.8	47.3±6.9	0.0013
12^th^ thoracic vertebrae	31.8±4.1	345.0±7.4	0.0171
Minimum true lumen width level	36.4±6.4	47.1±11.7	0.0045
Minor axis of the true lumen (mm)			
Proximal edge of entry	13.9±3.9	16.1±3.9	0.0956
Pulmonary artery bifurcated level	12.0±3.2	12.9±3.7	0.2071
12^th^ thoracic vertebrae	11.6±2.9	11.9±3.1	0.5687
Minimum true lumen width level	7.6±2.4	8.4±2.7	0.1933
Major axis of the true lumen (mm)			
Proximal edge of entry	32.6±3.7	34.8±7.0	0.1491
Pulmonary artery bifurcated level	28.4±5.4	31.6±6.8	0.0190
12^th^ thoracic vertebrae	24.2±3.3	25.9±3.8	0.0172
Minimum true lumen width level	25.9±3.4	29.9±4.1	0.0140

Diameter of the descending aorta and major axis of the true lumen enlarged significantly except the proximal edge of entry level. Minor axis of the true lumen did not enlarge in all levels.

Regarding the preoperative proximal neck anatomy, although no significant differences were observed in the proximal diameter between the two groups, the proximal neck length was significantly shorter in the narrow TL group, with the shortest length being 0 mm. Moreover, the number of patients with a proximal neck length of <15 mm was significantly higher in narrow TL group (**Table 3**). There were no significant differences between the two groups in terms of the type of device, proximal SG diameter, and frequency of the use of tapered-type device. Proximal oversizing was not significantly different between the two groups in cases SG was deployed in the artificial grafts. However, it was significantly smaller in the narrow TL group in patients whose proximal landing zone was native aorta. In the narrow TL group, SG was deployed from Zone 3 in all patients. On the other hand, the implantation site was Zone 3 in 14 patients (70%), Zone 2 in 5 patients (including 1 case of left common carotid artery–left subclavian artery bypass, 3 cases of stent implantation from the region of origin of the left subclavian artery, and 1 case of left subclavian artery simple closure), and Zone 1 in 1 patient in the aneurysmal group. Thus, the number of patients with a proximally implanted SG was significantly more in the aneurysmal group than in the narrow TL group. As for the patient with Zone 1 TEVAR in the aneurysmal group, SG was implanted in Zone 2 after left common carotid artery–left subclavian artery bypass. However, SG proximal edge extended to the just distal to brachial artery and the SG covered the origin of the left common carotid artery, the bared stent was implanted at the origin of the left common carotid artery. There was no significant difference in the frequency of proximal balloon attachment between two groups and all patient with proximal balloon attachment of SG for native aorta landing in narrow TL group underwent TEVAR before 2015. Three patients in the aneurysmal group had an additional proximal SG implantation for Type Ia endoleak. The operation time was significantly shorter in the narrow TL group. In the narrow TL group, Type Ia endoleak was detected by final intraoperative angiogram in seven patients (1 of 8 cases prior to 2015 (13%) and 6 of 12 cases (50%) after 2016). The frequency of conformable thoracic stent graft (CTAG) (W. L. Gore & Associates, Delaware, NA, USA) implantation was 2 out of 6 patients (33.3%) with Type Ia endoleak and 1 out of 6 patients (16.7%) without Type Ia endoleak in the narrow TL group after 2016. The frequency of intraoperative Type Ia endoleak was slightly higher in the narrow TL group, however, the frequency of definitive Type Ia endoleak did not differ between the two groups at 1 week and 6 months postoperatively (**Table 3**).

**Table 3:** Table 3 Proximal neck anatomy, device, operative procedure and the frequency of Type Ia endoleak

	Narrow TL	Aneurysmal	p value
	n=26	n=20	
Proximal neck			
Diameter (mm)	28.6±3.1	29.4±4.8	0.5549
Length (mm)	31.5±28.8	55.4±38.9	0.0078
Length<15 mm	10 (38.5%)	0	0.0024
TL diameter at entry/proximal neck diameter (%)	71.1±11.2	88.2±11.3	0.0002
Device			
CTAG	9 (34.6%)	10 (50.0%)	
TX2	5 (19.2%)	5 (25.0%)	
Variant/Navion	9 (34.6%)	2 (10.0%)	
Relay	3 (11.5%)	3 (15.0%)	0.2900
Device type			
Straight	24 (92.3%)	15 (70.0%)	
Taper	2 (7.7%)	5 (25.0%)	0.2127
Device diameter of proximal side (mm)	32.0±3.7	34.4±4.5	0.0599
Oversizing for proximal neck (%)			
Native aorta	111.1±7.0	116.1±3.8	<0.0001
Artificial graft	118.1±4.8	124.0±14.4	0.6171
Proximal Balloon attachment for the native aorta	6 (30.0%)	7 (53.8%)	0.1712
Additional Stentgraft deployment	0	3 (15.0%)	0.0751
Proximal landing zone			
Zone 1		1 (5.0%)	
Zone 2		5 (25.0%)	
Zone 3	26 (100%)	14 (70.0%)	0.0041
Operation time (minutes)	74±22	98±40	0.0087
Type Ia endoleak			
Final angiogram	8 (30.8%)	1 (5.0%)	0.0572
1 week after TEVAR			
Definitive Ia	1 (3.9%)	1 (5.3%)	
Possible Ia	3 (11.5%)	6 (31.6%)	0.2276
6 months after TEVAR			
Definitive Ia	0/18 (0%)	1/16 (6.3%) an	
Possible Ia	0/18 (0%)	4/15 (25.0%)	0.0184
1 year after TEVAR	0/6 (0%)	1/9 (11.1%)	0.9999

Proximal neck diameter was significantly shorter in Narrow TL group. The frequency of additional stentgraft deployment tended to be high in aneurysmal group and stentgraft was deployed proximal to one 3 for significantly more patients in aneurysmal group. The frequency of Type Ia endoleak tended to be high in Narrow TL group and the frequency of Type Ia endoleak was significantly higher in aneurysmal group 6 months after TEVAR.

Preoperatively, the proximal diameter ratio which represent the degree of proximal neck tapering, was significantly smaller in the narrow TL group (71.1%±11.2% in the narrow TL group vs. 88.2%±11.3% in the aneurysmal group). However, the proximal diameter ratio significantly increased in the narrow TL group (p<0.0001) 1 week after TEVAR, and there was no significant difference between two groups (95.9%±11.7% in the narrow TL group, vs. 90.8%±15.9% in the aneurysmal group). The proximal diameter ratio in the aneurysmal group did not change significantly from prior to 1 week after the TEVAR (p=0.4315).

**Figure 1:**
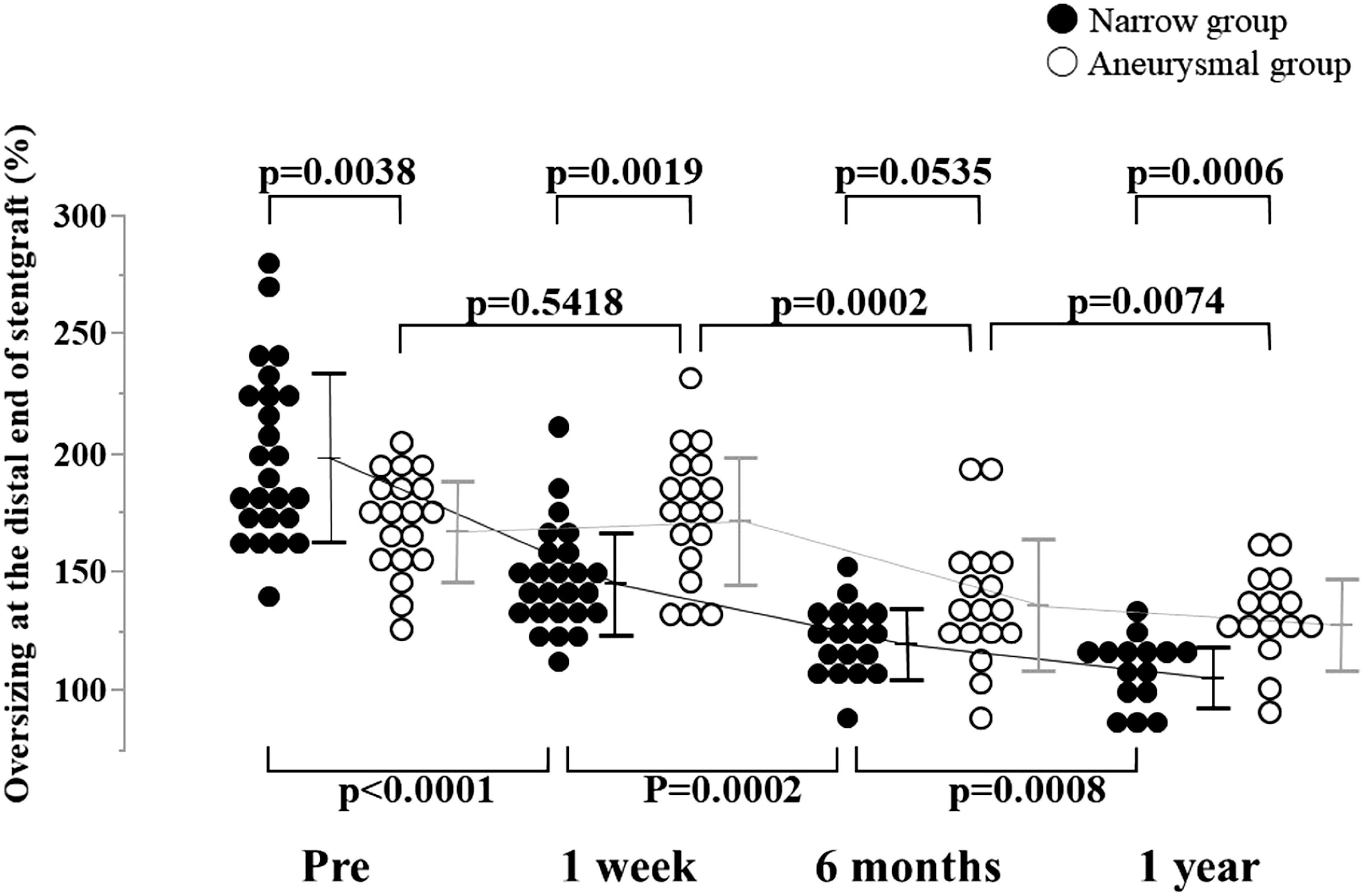
Fig. 1 Serial change of oversizing at the distal end of stentgraft. In narrow group, distal oversizing became significantly smaller 1 week after TEVAR and continued to become small until 1 year after TEVAR. In aneurysmal group, distal oversizing did not become small 7 days after TEVAR, then became small 6 months or 1 year after TEVAR. Distal oversizing was significantly larger in narrow group pre-TEVAR, however it became significantly smaller in narrow group 1 week after and 1 year after TEVAR.

**Figure 2:**
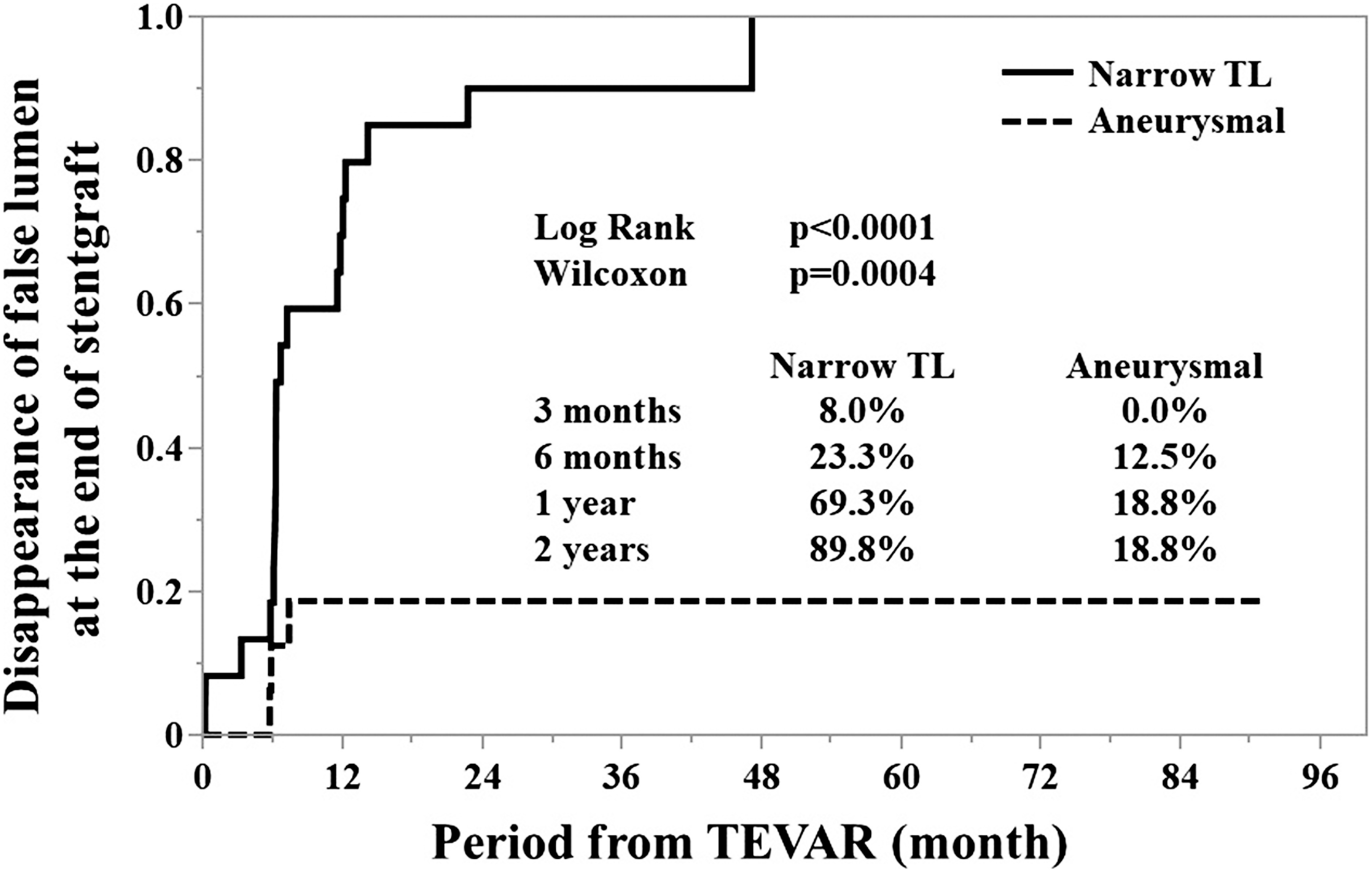
Fig. 2 Disappearance of false lumen at the end of stentgraft. False lumen at the end of stentgraft disappeared significantly earlier and in more patients in narrow group.

**Figure 3:**
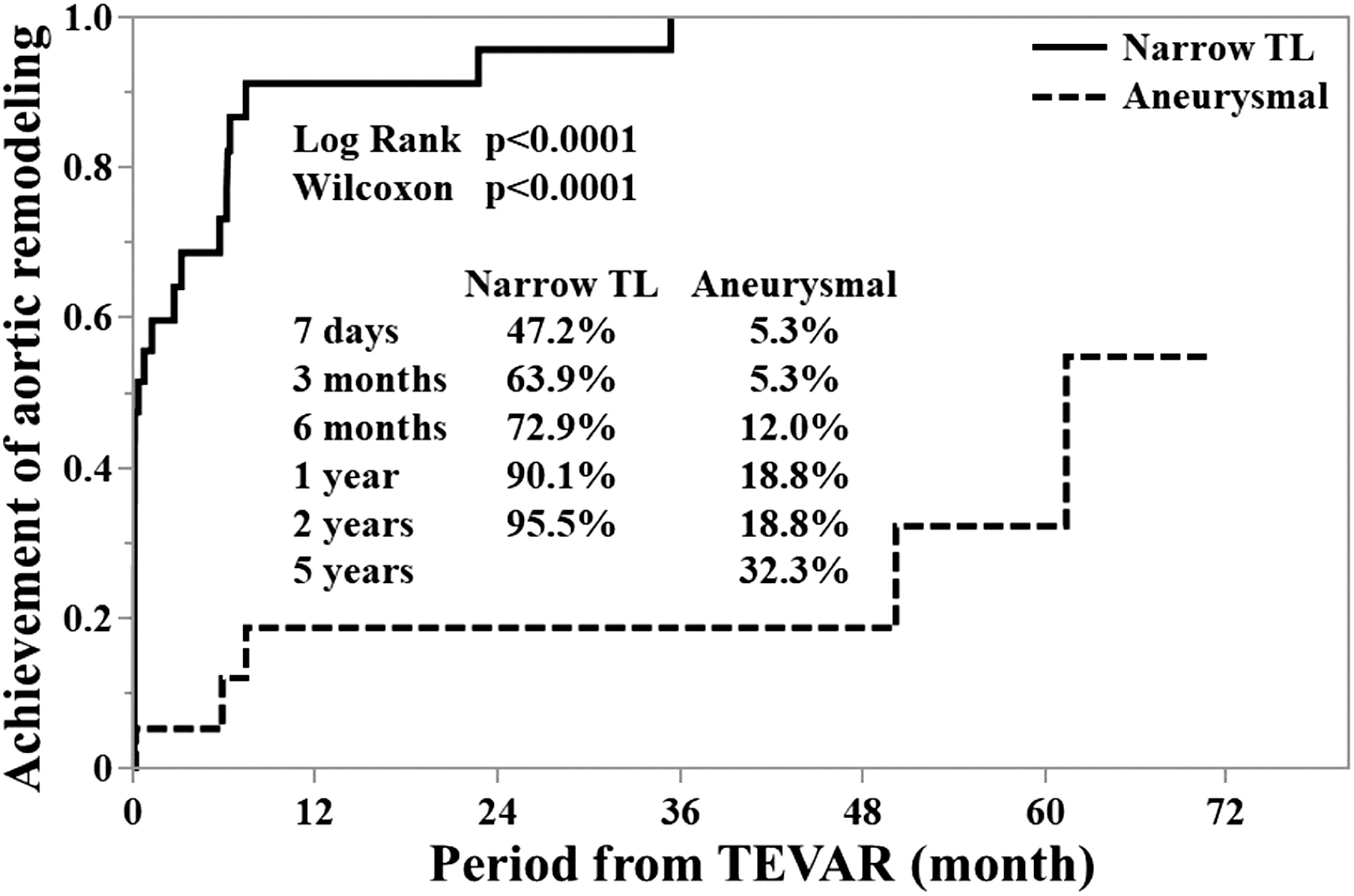
Fig. 3 Achievement of aortic remodeling. Aortic remodeling defined as true lumen diameter ≥50% of aortic diameter and closure of false lumen was significantly better in narrow group.

**Figure 4:**
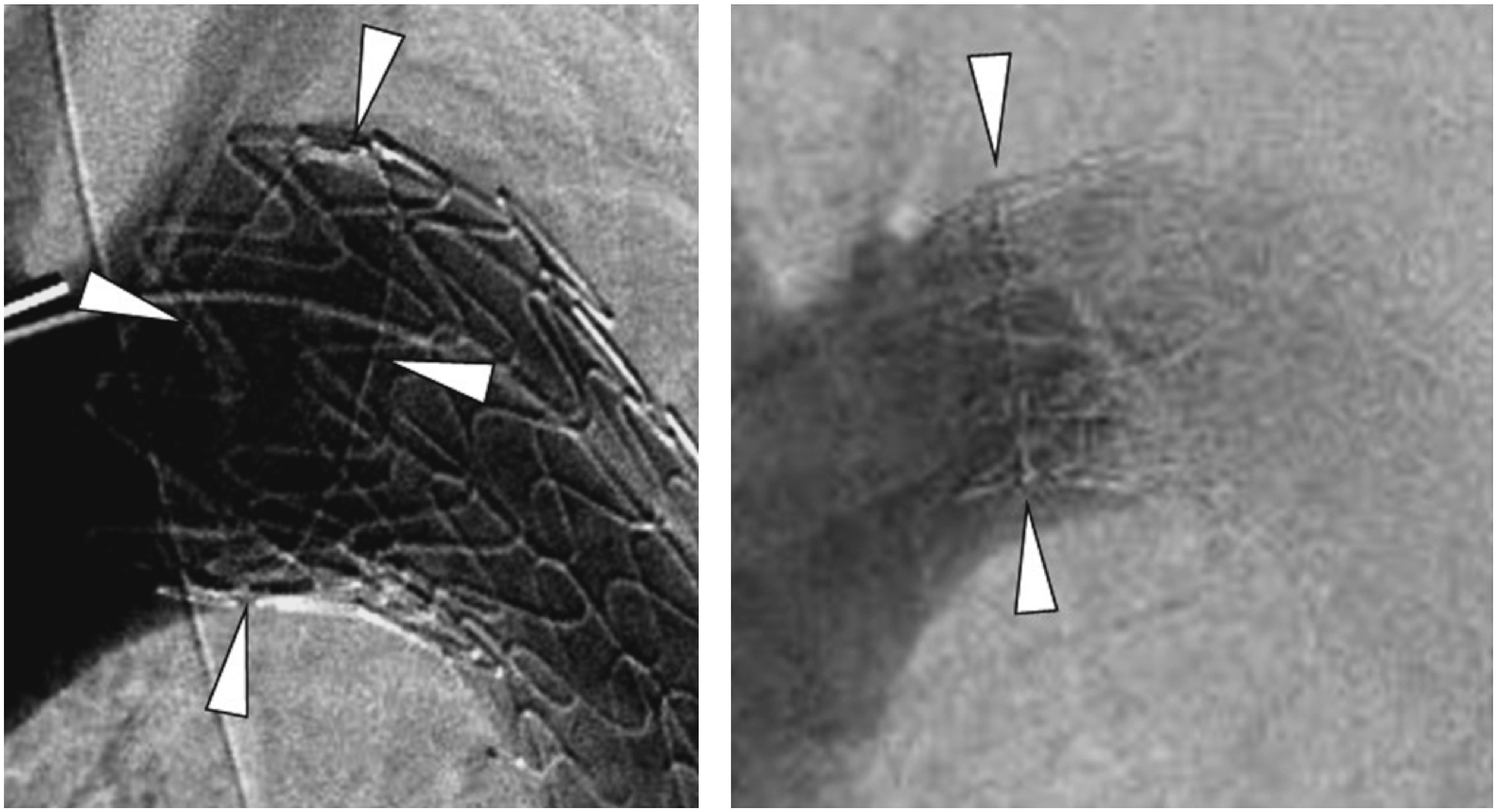
Fig. 4 Angiogram during stentgraft deployment of the patient with stentgraft migration in narrow group. Left panel: In this patient, primary entry existed just beneath the left subclavian artery and location of primary entry was posterior aspect of the aorta. CTAG active control was deployed just distal to the left subclavian artery. Proximal edge of stentgraft seemed to be oval shape as shown with white triangle. Right panel: In this patient, aortic diameter enlarged 6 mm three months after TEVAR, and GTAG active control was implanted just distal to the left carotid artery. At this time, proximal edge of stentgraft was visualized as perpendicular view at the half deployment. Proximal edge of stentgraft became straight line by final angiogram as shown with white triangle.

**Figure 5:**
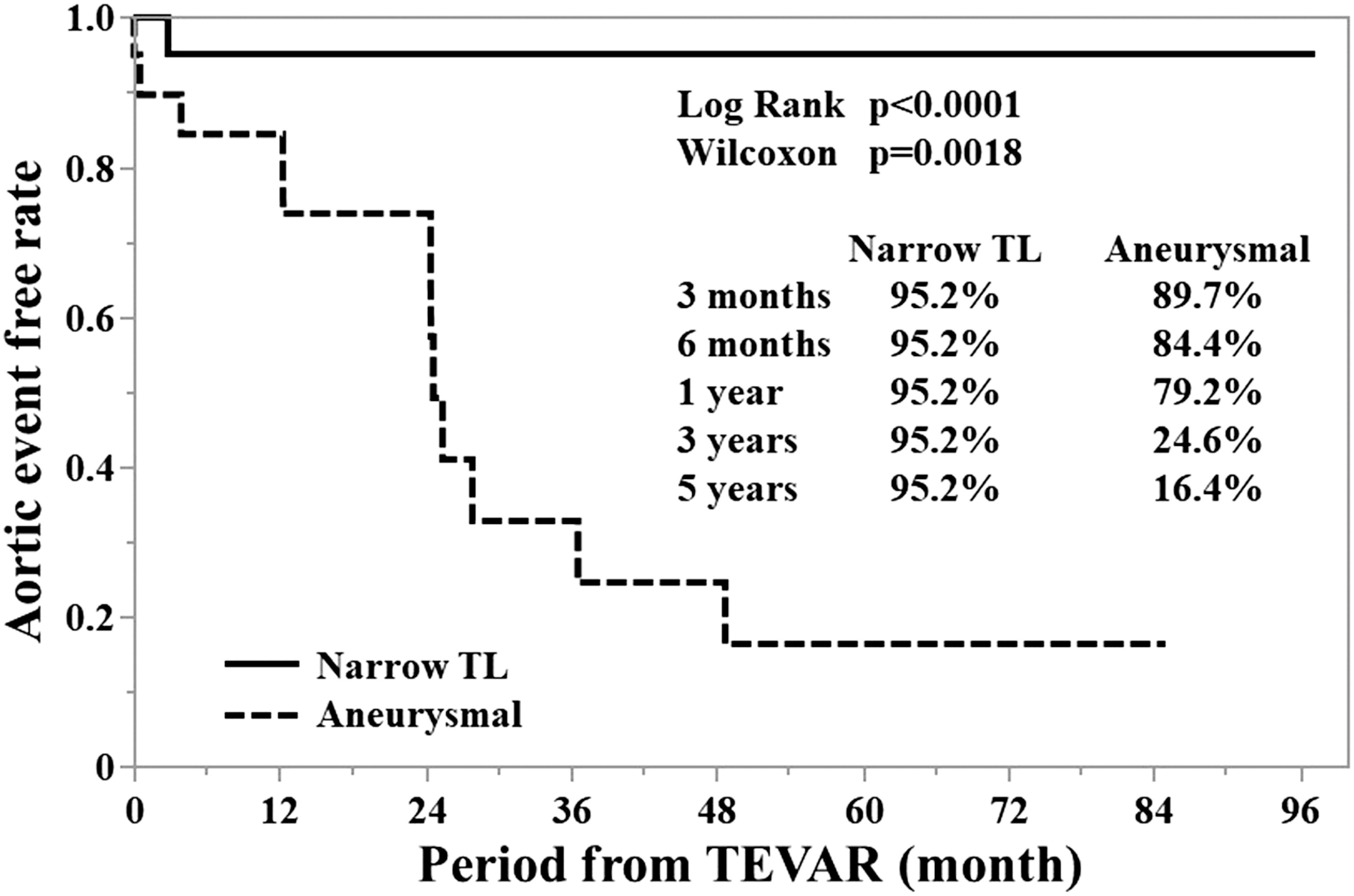
Fig. 5 Aortic event free rate. Dissection related death, retrograde Type A dissection, stentgraft induced new entry and aortic expansion 5 mm or more were defined as aortic event. Aortic event free rate was significantly better in narrow group.

Preoperatively, the TL ratio in the SG end that constituted the distal landing zone did not differ between two groups (40.6%±17.1% in the narrow TL group and 41.2%±18.8% in aneurysmal group, p=0.999). However, in the narrow TL group, it significantly increased to 65.0%±8.8% at 1 week, 90.0%±13.3% at 6 month and 97.4%±7.3% at 1 year after TEVAR (p<0.0001 at all postoperative time points compared with the preoperative value). In the aneurysmal group, the TL ratio changed to 42.7%±11.6% at 1 week (p=0.6495), 55.8%±18.9% at 6 months (p=0.2708) and 61.7%±20.3% (p=0.0431) at 1 year after TEVAR. Thus, no significant changes were observed until 6 months after TEVAR and the ratio significantly increased after 1 year after EVAR in the aneurysmal group. The postoperative TL ratio was significantly higher in the narrow TL group throughout the post TEVAR follow up period (p<0.0001 at all postoperative time points). The preoperative distal oversizing was significantly larger in the narrow TL group (198%±35% vs. 167%±21% in the aneurysmal group). However, distal oversizing decreased significantly in the narrow TL group at 1week after TEVAR and became significantly smaller than that of aneurysmal group (145%±22% in the narrow TL, vs. 172%±27% in the aneurysmal group). At 1 year after TEVAR, it remained to be significantly lower in the narrow TL group than in the aneurysm group (106%±13% vs. 128%±19%) (**Fig. 1**). The disappearance of the false lumen at the SG end occurred earlier and more frequently in the narrow TL group. The false lumen disappeared in 90% of the patients in the narrow TL group 2 years after TEVAR, whereas only 19% of the patients in the aneurysmal group experienced the disappearance of the false lumen (**Fig. 2**). Aortal remodeling was achieved earlier and more frequently in the narrow TL group (**Fig. 3**). Only one patient in the narrow TL group experienced an aortal event. In this patient, entry was just beneath the left subclavian artery in the posterior aspect of the aorta. CTAG active control was implanted just beneath the left subclavian artery, and TEVAR procedure was completed because TL dilatation was identified even Type Ia endoleak was detected by final angiogram. However, contrast-enhanced CT performed at 1 week after TEVAR revealed the SG distal migration and entry was not covered by SG. Also, true lumen dilatation was not observed. Contrast-enhanced CT performed at 3 months after the surgery revealed that the maximum aortic diameter had enlarged by >5 mm because of the dilation of the false lumen. In this case, the SG was implanted just beneath the left subclavian artery on the greater curvature side during TEVAR. However, proximal edge of SG was oval shape, instead of straight line by final angiogram as shown in **Fig. 4** and the proximal edge of the SG in the anterior or posterior part of the aorta may not have been situated just beneath the left subclavian artery; therefore, the entry posterior to the aorta may not be covered by the SG. In this case, CTAG active control was implanted proximally at 3.5 months after TEVAR procedure, and at that time, the SG was implanted just distal to the left common carotid artery to ensure that its proximal edge was visualized completely laterally, and, Type Ia endoleak disappeared (**Fig. 4**). In the aneurysmal group, postoperatively, the aortic events, including overlaps, were observed in one case of death due to a rupture of the descending aorta (after 3 days), two cases of RTAD (after 3 years in one case and 5 years in one case), five cases of stentgraft induced new entry (after 2 weeks in one case, 2 years in two cases, 3 years in one case, and 4 years in one case), and eight cases of aortic diameter dilation of ≥5 mm (after 4 months in one case, 1 year in three cases, 1.5 years in one case, and 2 years in three cases). Therefore, the aortic events free rate was significantly poorer in the aneurysmal group than in the narrow TL group (**Fig. 5**). As the risk factors of SINE, the duration from the onset of TBAD to undergoing TEVAR, the use of a tapered SG, the TL short diameter, the TL long diameter, oversizing at the distal part of SG, and the angle between the SG and aorta at the SG distal edge at 1 week after TEVAR were compared between the patients who developed SINE and those who did not. The duration from the onset of TBAD to undergoing TEVAR tended to be longer in patients with SINE (163±74 months in the patients with SINE and 86±60 months in those without SINE; p=0.0868) and the TL long diameter at the SG edge was significantly longer in patients with SINE (29.8±1.8 in patients with SINE and 27.1±4.2 in those without SINE; p=0.0295). However, no significant differences were observed in other factors. As the risk factors for the maximum aortic diameter dilation of ≥5 mm, the duration from the onset of TBAD to undergoing TEVAR, preoperative maximum diameter, and presence or absence of a patent false lumen in the thoracic aorta (cases with a Type Ia endoleak, including those with possible Ia, were considered to have patent false lumen) were evaluated. In cases with ≥5 mm dilation, the frequency of a patent false lumen tended to be higher at 1 week after TEVAR (62.5% in patients with dilation and 18.2% in those without dilation; p=0.0739) and significantly higher at 6 months (62.5% in patients with dilation and 0% in those without dilation; p=0.0256). No significant differences were observed in the other factors between the two groups.

## Discussion

The early mortality rate after conservative therapy for Stanford Type B aortic dissection is reported to be 8.3%–10%^1,2)^ and conservative therapy has been indicated for patients with TBAD without complications. However, the long-term prognosis of these patients remains poor, with the survival rate decreasing around 3 years after the onset of TBAD. The mortality rate after 4 years was reported to be 25%–40%,^2)^ and 28% of patients require some type of treatment after 5 years.^3)^ At our hospital, we introduced TEVAR for patients with narrow TL with minor TL axis of <50% of the aortic diameter at onset or during follow-up periods, and for patients with dissecting aortic aneurysms in 2012. Also, TEVAR has been performed in patients with ULP when the false lumen or maximum aortic diameter expansion, however, the patients with ULP were excluded from this study because there was not patent false lumen in proximal and distal landing zone usually in these patients. In this study, appropriateness of our device selection and TEVAR procedure were evaluated in the patients with patent false lumen.

The possibility that TEVAR for TBAD would promote the aortic remodeling and improve long-term prognosis was pointed out when we first introduced TEVAR for patients with narrow TL. However, until around 2010, there was a few evidence on the long-term survival benefit of TEVAR in such patients. According to the 2011 edition of the Guidelines for Diagnosis and Treatment of Aortic Aneurysm and Aortic Dissection,^4)^ TEVAR was recommended for chronic dissection with surgical indications (Class IIa) but TEVAR for acute dissection to prevent future aneurysmal dilatation was not recommended (Class IIb). However, the INSTEAD-XL Trial,^5)^ which investigated the five-year prognosis after TEVAR, reported that TEVAR improved prognosis and other study also reported survival benefit by surgical intervention for TBAD.^6)^ The concept of high-risk uncomplicated TBAD, in which the expansion of false lumen is anticipated among the TABD without complication, was introduced in the 2020 edition of the Guidelines for Diagnosis and Treatment of Aortic Aneurysm and Aortic Dissection^7)^ and the guidelines recommended that thoracic entry closure should be performed within 1 year of onset as preemptive TEVAR (Class IIa). The TEVAR performed for narrow TL patients at our hospital can be considered preemptive TEVAR as per the 2020 guidelines. Preemptive TEVAR is performed for the patients without actual false lumen dilation, therefore, (1) accurate selection of cases with false lumen dilation in future, (2) performing TEVAR without complications, and (3) achieving favorable aortic remodeling, would be important.

According to the guidelines,^7)^ predictive factors for chronic false lumen dilation include age, heart rate, underlying disease, and fibrin degradation product level. Moreover, the anatomical risk factors for false lumen dilation include aortic diameter of ≥40 mm, oval-shaped TL or round-shaped false lumen, patent false lumen, single entry, false lumen width of ≥22 mm, and large entry of ≥10 mm. Preventive factors for false lumen dilation include aortic diameter of <40 mm, round- or oval-shaped TL, and complete thrombus occlusion of the false lumen. We thought that intimal flap would be soft during the acute phase following onset^8)^ and the TL shape was defined by the balance of blood flow or blood pressure of the TL to that of the false lumen. In that case, the short diameter of TL would be 50% or less of aortic diameter when blood flow or blood pressure of false lumen is higher than those of TL in acute phase, and the false lumen would expand without entry closure in such patients.^9)^ Based on this consideration, out indication of preemptive TEVAR has been the minor axis of the TL is <50% of the aorta.

As the preemptive TEVAR procedure, it is important to prevent possibly fatal RTAD in the proximal landing zone and to avoid Type Ia endoleak to achieve favorable aortic remodeling. The risk factors for RTAD include TEVAR for the patients with aortic dissection, particularly during the acute phase of aortic dissection, and the excessive SG oversizing. Whether the use of proximal bared SG devices increases the likelihood of RTAD is a controversial topic.^10–13)^ In this study, we used a device with a proximal diameter of 105%–115% oversizing for cases with the native aorta constituted the proximal landing zone. The type of device was selected with the aim of preventing a Type Ia endoleak based on the proximal anatomy and the device with proximal bared SG was selected for 13 patients (48%) in the narrow TL group. Regarding the implantation procedure on the proximal side, the guidelines^7)^ recommend that the proximal landing edge should be of >20 mm proximally from the proximal entry edge, and the proximal landing site should be non-dissected with an aortic diameter of ≤37 mm. In cases requiring a device of ≥40 mm, we replace the aorta instead of TEVAR because we think the patient with large aorta is not the candidate for TEVAR. Therefore, TEVAR is indicated for cases with an aortic diameter of ≤37 mm in the proximal landing zone in our hospital. However, for the proximal neck length, we selected Zone 3 as the implantation site for all patients, assuming that entry closure will be achieved if the SG is implanted just beneath the left subclavian artery. Initially, balloon attachment of SG was performed on the proximal side when an antegrade contrast enhancement was detected in the false lumen by angiogram. However, we encountered a case in which aortic remodeling was observed by contrast-enhanced CT 6 months after TEVAR despite Type Ia endoleak was detected intra-operative final angiogram. After performing TEVAR in eight patients with narrow TL in 2016, we redefined the endpoint as TL dilation instead of disappearance of Type Ia endoleak in narrow TL group. According to this new therapeutic strategy, TEVAR should be terminated even when an antegrade false lumen contrast effect is detected on final contrast-enhanced imaging, if TL expansion was confirmed. Using this therapeutic strategy, entry closure was achieved within 6 months postoperatively even in case where the distance from left subclavian artery to the entry was only 3 mm, except for one case where the dissection extended into the left subclavian artery and entry was in the left subclavian artery. The reason why favorable aortic remodeling could be obtained with zone 3 TEVAR in narrow TL group would be (1) tapered proximal landing zone preoperatively became almost straight within 1 week after TEVAR because the TL diameter at the entry site where the SG was implanted dilated rapidly and (2) the proximal neck area regained nearly normal aortic anatomy because of favorable aortic remodeling. In a case wherein entry closure was not achieved by Zone 3 TEVAR, CT performed 1 week after TEVAR revealed that the SG had migrated to the distal side and SG did not cover the entry. In this case, SG proximal edge was not visible laterally during implantation, as shown in **Fig. 4**, and the aortic remodeling in proximal landing zone could not be obtained because entry existed in the posterior aspect of the aorta was not covered with SG. In transcatheter aortic valve implantation, setting the fluoroscopic angle to obtain a perpendicular view that laterally visualizes the aortic valve annus and device edge is critical. In this case, the angle required to laterally visualize the tip was set at the timing of the half-deployment when CTAG active control was additionally implanted at 3 months after TEVAR (**Fig. 4**). This suggests the importance of SG deployment not only in the frontal view of the aortic arch but also in the completely lateral view of the proximal edge of SG when proximal neck length is short or entry existed just distal to the left subclavian artery. In 2016, we decided not to perform balloon attachment of SG when TL dilatation was confirmed by intraoperative angiogram, even Type Ia endoleak was detected by angiogram. This policy was applied for CTAG for which balloon attachment is recommended after implantation. With this TEVAR strategy, TEVAR was terminated with residual Type Ia endoleak by final angiogram in 50% of the cases, and, the Type Ia endoleak disappeared within 6 months postoperatively in all cases, except in one case where SG migration occurred. Therefore, our proximal implantation strategy since 2016 would be appropriate.

Preventing SINE is thought to be important for distal landing. Moreover, some studies have reported that excessive distal oversizing is a risk factor for SINE.^14–16)^ In the guidelines,^7)^ 110%–120% oversizing for SG diameter is recommended; accordingly, the use of either a tapered graft or two SGs are encouraged. Because it was unclear how the distal anatomy would change in patients with narrow TL when TEVAR was started in 2012, a tapered graft was occasionally selected in our early experience. However, we changed our approach in 2016 to allow the distal oversizing of 200% and chose a straight-type device, in principle, after noticing that the TL on the SG edge dilated after 1 week of surgery and that it decreased oversizing. In the present study, the TL diameter in the distal landing zone became 90% of the aortic diameter at 6 months postoperatively, and distal oversizing became 106% at 1 year after TEVAR. Moreover, among the cases with a patent false lumen in the distal landing zone, the false lumen disappeared in approximately 70% and 90% of the cases at 1 and 2 years after TEVAR, respectively, and the aorta of distal landing zone became to be the almost normal aorta. According to the abovementioned findings, the proximal landing zone was nearly normalized at 1 week after TEVAR and distal landing zone became almost normal over time in the narrow TL group. Thus, TEVAR for TBAD became healthy-to-healthy TEVAR, which is the principle of SG treatment, within 2 years in many narrow TL group patients, and a favorable long-term outcome can be expected from this procedure. Although it is impossible to reach a definitive conclusion given the small sample size of this study, the choice of devices and implantation procedure appears to be valid to some extent as no patients in the narrow TL group developed RTAD or distal SINE and the aortic remodeling achievement rate and aortic-event free rate were significantly better than those in the aneurysmal group.

Meanwhile, in the aneurysmal group, an SG was implanted from a site that was more proximal than Zone 2 in>30% of the cases to sufficiently secure the proximal landing zone, and proximal neck length of ≥15 mm (≥20 mm, except for one patient) was secured in all patients. The average proximal oversizing of the device was 116%. The intraoperative endpoint of TEVAR was defined as disappearance of Type Ia endoleak, and an additional SG was implanted proximally, if needed during the study period, and TEVAR was terminated with disappearance of a Type Ia endoleak by final angiogram in all patients, except for one patient. Postoperatively, the incidence of Type Ia endoleak evaluation by contrast-enhanced CT was difficult, because when the influx of contrast agent into the false lumen is continuous from the proximal SG implantation site to the distal false lumen, this could be Type Ia endoleak or contrast effect from the distal re-entry. In this study, a definitive Type Ia endoleak was observed in only one patient at 1 week and 6 months postoperatively. This result suggested that our selection of devices and implantation procedure for the proximal landing zone would be appropriate. However, distal SINE was observed in five patients (26%). Although no significant differences in distal oversizing were observed between the SINE and non-SINE groups, the selection of devices with an average distal oversizing of 166% was itself possibly inappropriate. When TEVAR is performed 5 years or more after the onset of TBAD, several measures, such as using multiple SGs, using one or more tapered devices, implanting a small diameter SG in the distal SG edge,^17)^ and preventing damage on the aorta by SG at the distal SG edge using the petticoat technique,^18)^ should be considered. Additionally, there were eight cases of maximum diameter dilation of ≥5 mm, and the frequency of residual false lumen contrast effect was significantly higher in these cases than that in those with non-dilated lumen at 6 months postoperatively. The maximum diameter would increase when there is residual blood flow into the false lumen from the TL via the proximal entry or the distal reentry. Therefore, to completely stop blood flow into the false lumen, some maneuver such as the distal reentry closure, candy plug implantation that blocks blood flow into the false lumen,^19)^ the Knickerbocker technique^20)^ or petticoat technique,^18)^ or implanting an SG that covers a wider segment^10)^ should be considered.

As endovascular treatment for TBAD with a dissecting aortic aneurysm is not recommended (Class IIb),^7)^ it is crucial to popularize the concept of preemptive TEVAR in the future. The guidelines do not specify the optimal timing for preemptive TEVAR and only stated as “optimal timing of preemptive TEVAR would be within 6 months of onset.” The VIRTUE Registry^10)^ and Li et al.^11)^ reported the results of TEVAR performed at different period after TBAD onset. In the VIRTUE Registry,^10)^ 15 cases of acute TBAD undergoing TEVAR within 14 days after the onset of TBAD, 24 cases of subacute TBAD undergoing TEVAR after 15–92 days, and 26 cases of chronic TBAD undergoing TEVAR after ≥93 days were evaluated. Of these, 6 cases of acute TBAD died within 30 days; however, no cases of subacute and chronic TBAD died within 30 days. Although no significant differences in the achievement rate of aortic remodeling were observed between the acute and subacute groups, the rate was significantly lower in the chronic group than in the other two groups. Similarly, in 2020, Li et al.^11)^ reported the results of a study involving 165 cases of acute TBAD undergoing TEVAR within 14 days after the onset, 111 cases of subacute TBAD undergoing TEVAR after 15–90 days, and 33 cases of chronic TBAD undergoing TEVAR after ≥91 days. The patient background revealed that many patients in the acute group had dissection-related complications, whereas many patients in the chronic group developed false lumen dilation. The perioperative treatment results showed that the major complications rate and the retreatment rate were significantly lower in the subacute group than in the other two groups. Also, long-term outcome, major complication rate and that including death was significantly better in the subacute group than other 2 groups. Moreover, the overall mortality rate and the rate of dissection-related deaths tended to be lower in the subacute group. Meanwhile, the disappearance rate of the false lumen was significantly lower in the chronic group. These reports suggest that it is safe to perform preemptive TEVAR at least 2 weeks after onset in patients with TBAD without complications. Factors affecting the remodeling of the thoracic aorta include the timing of TEVAR as well as the presence or absence of blood flow into the false lumen from the distal reentry. Notably, some patients achieved aortic remodeling after undergoing TEVAR at up to 22 months after the onset of TBAD in our study, as well as Saiki et al. reported that 50% of the patients who underwent TEVAR within 1 year after onset achieved remodeling.^17)^ Preemptive TEVAR should be performed when the plasticity of intimal flaps is maintained to facilitate the dilation of the TL and to promote the false lumen thrombosis and disappearance. According to a study that investigated aortic anatomy and pathological changes after the onset of TBAD,^8)^ intimal flaps thickened at a rate of 1.2 and 0.41 mm/year during the acute and subacute phases, respectively and this thickening stabilized 83 days after the onset of TBAD. Histologically, the fragmentation of elastin and the consequent increase in fibrosis correlates with the decreased motility and thickness of the intimal flaps, which suggested fibrosis in intimal flaps may not be advanced in the subacute phase within 3 months of onset. Moreover, both the VIRTUE Registry^10)^ and Li et al.^11)^ reported that favorable remodeling was achieved when TEVAR was performed within 3 months after the onset of TBAD. Therefore, it is highly expected to achieve favorable aortic remodeling by the preemptive TEVAR within 3 months after onset.

The limitation of this study are as follows, the sample size was small, the TEVAR procedure for the narrow TL group was changed during the study period, distal oversizing should be 100%–110% and some maneuver to prevent blood flow into the false lumen should be considered to improve the outcome in the aneurysmal group. Further studies with more TBAD patients underwent TEVAR with new device selection policy and new TEVAR procedure would be required to evaluate the safety and long-term results of TEVAR.

## Conclusion

By the TEVAR performed during 2 weeks to 3 months after the onset with the policy such as a straight-type device with 105%–115% oversizing on the proximal side selection, Zone 3 TEVAR, TL expansion as the endpoint of TEVAR, favorable aortic remodeling was obtained without causing any perioperative complications or aortic events. In cases with a dissecting aortic aneurysm, TEVAR was performed with 120% oversizing on the proximal side, with the secured proximal landing zone of ≥20 mm and balloon attachment of SG or additional proximal SGs used intraoperatively, as necessary, a Type Ia endoleak could be prevented. However, when devices with an average oversizing of 166% in the distal landing zone were selected, the incidence rate of SINE was found to be 28%. Therefore, excessive oversizing should be avoided. If there is residual blood flow in the false lumen, the risk of 5 mm or more dilation in the aortic diameter increases. Hence, some maneuver should be taken to prevent blood flow into the false lumen as well as to avoid proximal entry closure. TEVAR for the patients with narrow TL during follow-up period was safe and simple procedure, and good long-term outcome is obtained. In contrast, in cases with dissecting aortic aneurysms, the TEVAR procedure became complicated, the frequency of secondary treatment increased, and long-term outcome was poor. In the future, preemptive TEVAR should be actively performed for patients with high-risk uncomplicated TBAD, including those with residual dissection in the descending aorta following surgery for Type A acute dissection.

## Supplementary Note

This study was approved by the Human Experimentation Ethics Committee at Showa University (acceptance number: 3125). The content of the study was presented at the 48th Academic Conference of the Japanese Society for Vascular Surgery (November 28, 2020).

## Notes

This is secondary publication from Jpn J Vasc Surg 2021; **30**: 347–357.
